# Boosting 2D Black Phosphorus Ambient Stability: Noncovalent Functionalization Using Viologen Molecules

**DOI:** 10.1002/smll.202410300

**Published:** 2025-03-21

**Authors:** Ishan Sarkar, Cong Guo, Cheng Peng, Yu Wang, Yafei Li, Xiaoyan Zhang

**Affiliations:** ^1^ Department of Chemistry and Chemical Engineering Chalmers University of Technology Kemigården 4 Göteborg SE‐412 96 Sweden; ^2^ Jiangsu Collaborative Innovation Centre of Biomedical Functional Materials School of Chemistry and Materials Science Nanjing Normal University Nanjing 210023 P. R. China

**Keywords:** ambient stability, black phosphorus, density functional theory, non‐covalent functionalization, viologen derivatives

## Abstract

Black phosphorus nanosheets (BPNSs) have recently emerged as a valuable addition to the diverse family of 2D materials, holding promises for a wide range of applications. However, their practical use is limited by poor stability under ambient conditions, as they degrade quickly when exposed to light, air, or moisture. Noncovalent functionalization offers a promising approach to address these challenges. Herein, viologen derivatives are incorporated into a BPNS suspension in acetonitrile, resulting in the formation of two hybrid materials. These hybrids are subsequently stored under ambient conditions to track their degradation over time. The degradation behavior of these functionalized BPNSs is analyzed and compared to that of pristine BPNSs stored in both nitrogen and ambient environments, using X‐ray photoelectron spectroscopy. Interestingly, the two viologen‐based hybrid systems exhibited varying degrees of ambient protection efficiency, attributed to differences in their average adsorption energies and aggregation kinetics with BPNSs. Methyl viologen‐functionalized BPNSs showed markedly reduced degradation in ambient conditions, with less pronounced differences for samples stored in a protected environment. This study introduces a promising strategy for enhancing the stability of BPNSs, making them more resistant to decomposition and potentially suitable for energy storage applications and optoelectronic devices.

## Graphical abstract

Viologen derivatives are employed to tackle BPNS oxidation challenges through a noncovalent strategy. Comprehensive characterization techniques, complemented by DFT calculations, provide detailed insights into these interactions. Notably, XPS analysis reveals that MV‐BPNS hybrids experience minimal oxidation under ambient conditions, highlighting the enhanced stability and unlocking the potential for advanced energy storage and next‐generation optoelectronic applications.

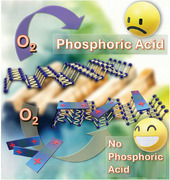

## 1 Introduction

Over the past decade, 2D materials including graphene, transition metal dichalcogenides, and BPNSs, have attracted significant attention from researchers worldwide.^[^ ^1^, ^2^, ^3^ ^]^ Their ability to manifest characteristics of the quantum realm in macroscopic dimensions has paved the way for modern technology.^[^ ^4^, ^5^ ^]^ Among all of them, 2D BPNSs, being relatively recent and having distinctive properties such as thickness dependent tunable direct band gap,^[^ ^6^, ^7^ ^]^ high charge‐carrier mobility^[^ ^8^ ^]^ and strong in plane anisotropy,^[^ ^9^ ^]^ have found inherent applications in photocatalysis,^[^ ^10^, ^11^, ^12^ ^]^ energy storage,^[^ ^12^, ^13^ ^]^ semiconductor‐electronics,^[^ ^12^, ^14^, ^15^ ^]^ optoelectronics,^[^ ^12^, ^16^, ^17^ ^]^ sensing and biomedicines.^[^ ^18^ ^]^

**Figure 1:**
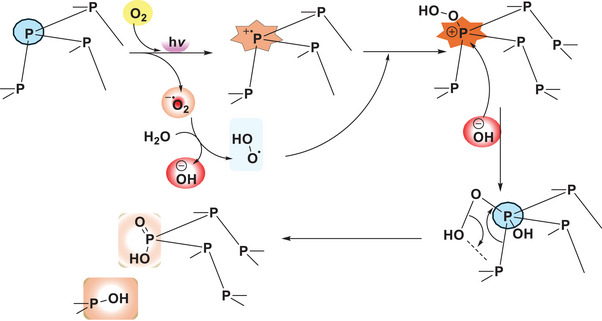
Plausible degradation mechanism of BPNSs under aerobic and light exposure.

Despite the extraordinary characteristics, the practical relevance of BPNSs is significantly limited by their poor stability under ambient conditions, primarily due to chemical degradation when exposed to light, air, and moisture.^[^ ^19^ ^]^ The high affinity of phosphorus atoms for oxygen, driven by the strong bond‐dissociation energy, accelerates this degradation process. It is hypothesized that degradation begins with a light‐dependent initiation process, where the nanosheets generate superoxide radical anions, leading to the formation of peroxide linkages (**Figure** **1**) on the nanosheets. This is followed by a propagation process, in which surrounding water molecules interact, ultimately resulting in the formation of phosphoric acid.^[^ ^20^, ^21^ ^]^

To date, some efforts have been made to address the issue of ambient instability by mitigating one or more of the three factors contributing to the degradation process, generally categorized as covalent and noncovalent approaches, depending on whether chemical bonding is involved.^[^ ^22^ ^]^ Each passivation method offers distinct advantages and trade‐offs, with the choice depending on the target application—such as optoelectronics, sensing, or field‐effect transistors (FETs)—and the specific type of 2D material involved. The properties of hybrid nanosheets after passivation depend on the substrate and the intended effect of the process. Covalent functionalization has provided itself as a promising strategy for stabilization with new and improved physicochemical properties. Surface modification and passivation of BPNSs via covalent bond formation can be performed through diazonium salt chemistry as shown by Ryder et al.^[^ ^23^ ^]^ Porphyrin functionalized BPNSs prepared though diazonium chemistry has provided significant protection against ambient degradation along with interesting photophysical properties.^[^ ^24^ ^]^ However, diazonium chemistry also carries the risk of a single‐electron transfer process, where electrons from the nanosheets transfer to the charged nitrogen species in diazonium salts. This leads to a high density of unpaired electrons on the nanosheet surface, ultimately causing interface amorphization as the system minimizes energy, resulting in red phosphorus‐like structures.^[^ ^25^ ^]^ Aryl‐iodonium salts^[^ ^26^ ^]^ and nucleophilic reagents^[^ ^27^ ^]^ also open a vast field of covalent modification. Azide based functionalization methods have been developed, which involve the formation of nitrene intermediates to establish P═N bonds. This approach has demonstrated significant improvements in ambient stability for the BPNSs.^[^ ^28^ ^]^ Wang et al.^[^ ^29^ ^]^ showed that when BPNSs are functionalized covalently using graphitic carbon nitrides, the P─N bonds can help regulating electron transfer processes from BPNSs to the carbon nitride moieties. This functionalization enables their use as controlled oxygen electrocatalysts and facilitates their utility as valuable components in zinc–air batteries (ZABs). Kuchkaev et al. showed that P─C bonds can directly be formed through carbene generation from chloroform by strong bases, e.g., potassium tert‐butoxide.^[^ ^30^ ^]^ Compared to non‐covalent functionalization, covalent functionalization was performed at lower concentrations to obtain a lower degree of functionalization, thus minimizing the detrimental increase in off current.^[^ ^23^ ^]^ A moderate level of covalent functionalization can potentially enhance the stability and optimize the properties of BPNSs.^[^ ^17^ ^]^

Non‐covalent functionalization leverages van der Waals interactions to passivate the conduction band of BPNSs, effectively halting the formation of superoxide radicals.^[^ ^17^ ^]^ Redox‐active electron‐deficient hydrophobic anthraquinone has been employed by Pumera and co‐workers^[^ ^31^ ^]^ to interact with BPNSs through van der Waals interactions, thus enhancing the charge storage capacity of the hybrid material. AlO_x_, initially used to passivate the BPNS surface and enhance the nanosheets' mobility and on/off ratio over time,^[^ ^20^ ^]^ was later found to increase the barrier potential for charge diffusion and electromigration at the grain boundaries when applied to graphene, leading to improved conductivity under high‐current treatment.^[^ ^32^ ^]^ Ag^+^ ions have been deployed to interact with the bare lone pairs of BPNSs through noncovalent interactions, which can enhance both the ambient stability and transistor performance of the exfoliated BPNSs.^[^ ^33^ ^]^ Previous theoretical calculations by Zhao et al. demonstrated that the self‐assembly of perylene‐3,4,9,10‐tetracarboxylic dianhydride on BPNS surfaces via van der Waals interactions can effectively shield the surface from oxidation while preserving the electronic band structure of BPNSs.^[^ ^34^ ^]^ This essentially implies that non‐covalent modification has a significantly smaller impact on the electronic structure of the nanosheets.

**Figure 2:**
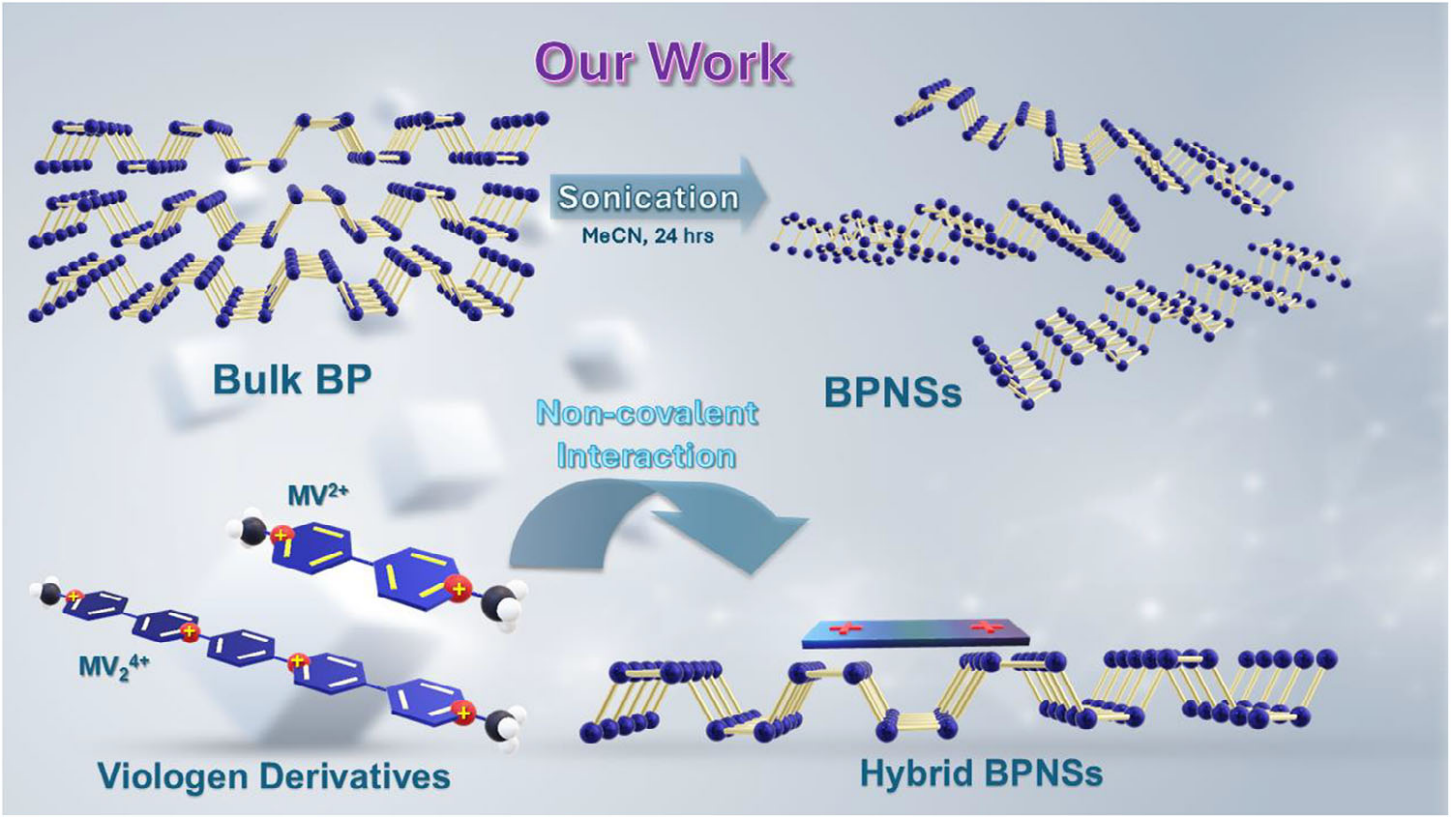
Schematic representation of non‐covalent functionalization between BPNSs and viologen derivatives.

Viologens and their derivatives are a class of molecules derived from 4,4′‐bipyridylium salts, characterized by rigid *π*‐electron deficient structures. They are well‐known for their exceptional reversible redox properties, which are crucial for various energy storage applications.^[^ ^35^ ^]^ The term “viologen” is derived from their capacity to produce a violet color upon reduction. The reduction process of viologens, which possess an extended *π*‐system, typically involves multiple radical‐cation intermediates that can be resonance‐stabilized.^[^ ^36^ ^]^ The radical cation generated upon reduction is intensely colored which makes it an outstanding candidate for electrochromic systems. Herein, two viologen derivatives with extended π‐systems have been employed to passivate the electronically dense surface of the BPNSs (**Figure** **2**).

## 2 Results and Discussion

**Figure 3:**
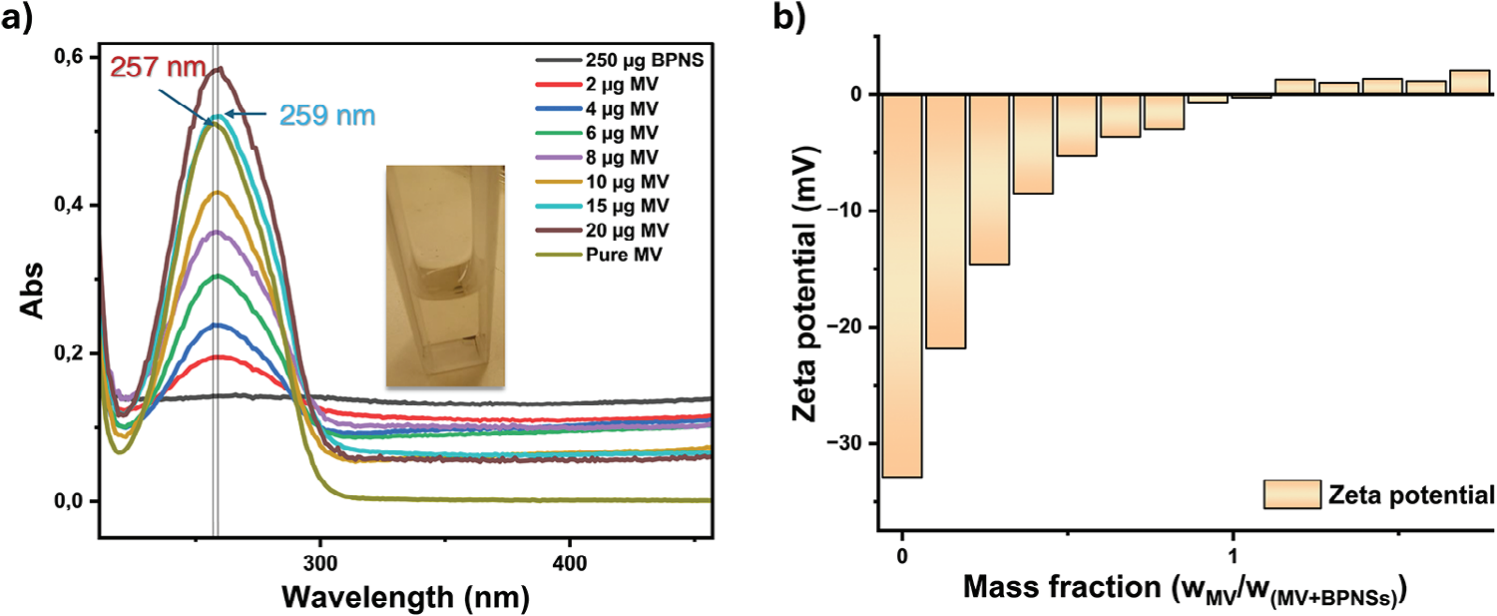
Titrimetric studies indicate the interaction between the MV molecules and BPNSs. a) UV–vis absorption and b) Zeta potential measurements. The absorbance of a 3.01 mL solution containing 0.0138 mg mL^−1^ of MV(PF_6_)_2_ in acetonitrile was measured for comparison. Zeta potential measurements were performed by adding MV molecules to BPNSs dispersed in water.

To test the hybrid formation, titration of the BPNSs by viologen derivatives were performed in acetonitrile and the experiments were monitored by UV–vis absorption spectroscopy. The peak molecular absorbance in acetonitrile was recorded at 256 nm for methyl viologen (MV). Red shifts in the absorption peak to 259 nm were observed for the MV samples (1 mg mL^−1^) with the gradual addition of a fixed amount of BPNSs (1.75 mL of 0.143 mg mL^−1^ dispersion), as shown in **Figure** **3**. The shift of the absorption peak suggests that there might be interactions between the MV molecules and BPNSs in the hybrid material, which can further be verified by the formation of precipitates. During titration, the addition of the molecular solution induces aggregate formation, altering the pre‐existing scattering behavior of BPNSs. After normalizing the peak absorbance in the range of 214–330 nm, the peak shift persists without significant broadening, as shown in Figure S4 (Supporting Information). The molecular absorption offset was similar to that of the hybrids, indicating that the broadening seen in the unprocessed plots was likely due to scattering from the hybrid aggregates. It is noteworthy that, when scaled up, the precipitation rate for the MV‐BPNS hybrid remained consistent. The Zeta potential study presented further insight on the charge‐neutralization point of BPNSs when the MV molecules (13.2 mg mL^−1^, 10 µL each time) were gradually added to a fixed amount of a BPNS dispersion (0.29 mg mL^−1^, 700 µL) in water. The zeta potential of the BPNSs was observed to be −33 mV when no titrant was added, however, upon addition of the MV molecules to the BPNSs, the zeta potential was observed to shift toward less negative value. The saturation point was observed at a mass fraction of 0.86 for MV (Figure 3) where the zeta potential within the hydrodynamic radius of the hybrids was mostly neutralized. More addition of the MV molecules results in slightly positive values and at the mass fraction of 0.89 for MV, the zeta potential was observed to be +2.1 mV.

**Figure 4:**
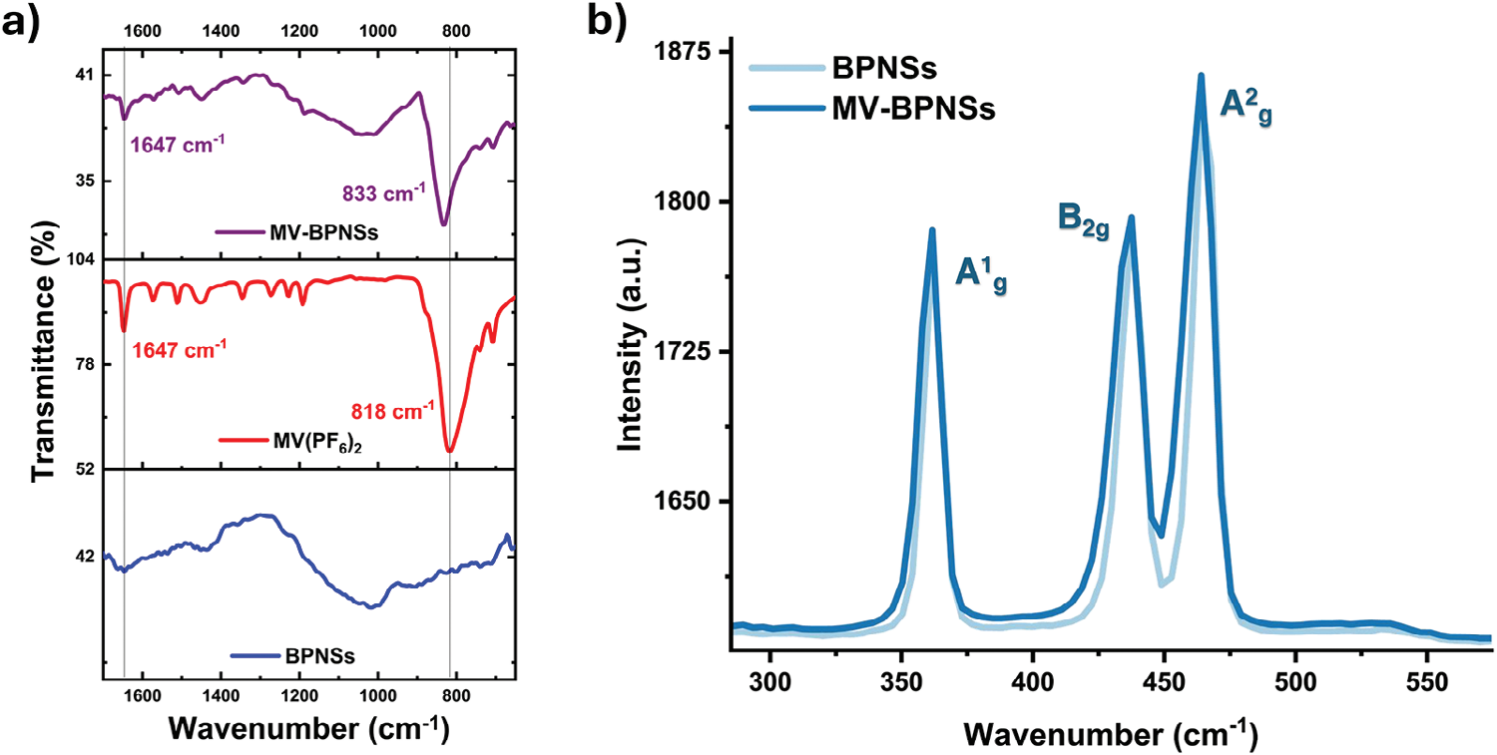
Studies indicate the interactions between the MV molecules and BPNSs. a) ATR‐IR, b) Mean Raman spectra of BPNSs and MV‐BPNSs measured using a 532 nm laser excitation. Blue shift in the IR for the out‐of‐plane bending modes at 818 cm^−1^ from the terminal methyl groups of the MV molecules suggests the possible interactions between the two components in the hybrid.

FTIR spectra were recorded for the MV and was compared to pure BPNSs and the MV‐BPNS hybrid by employing attenuated total reflectance (ATR) measurement. The C–H out‐of‐plane bending vibration of the terminal methyl groups can be observed at 818 cm^−1^ for MV, which is absent in the spectrum for the bare BPNSs. In the hybrid material, the out‐of‐plane bending mode shifted to 833 cm⁻¹ (**Figure** **4**), indicating a restriction in the MV's out‐of‐plane bending modes due to its stacking on the BPNS surface. The shift in the FTIR spectra suggests potential interactions between the MV molecules and BPNSs.

Mean Raman spectra were recorded using a 532 nm excitation laser and the mean Raman spectra was obtained by averaging the data thus obtained. The BPNSs show three prominent sharp peaks which correspond to the out‐of‐plane symmetric phonon mode A^1^_g_ at 362 cm^−1^, the in‐plane modes B_2g_ at 437 cm^−1^ and A^2^_g_ at 468 cm^−1^.^[^ ^24^, ^37^, ^38^ ^]^ After noncovalent functionalization, all the peaks for their respective vibrational modes were preserved in the MV‐BPNS hybrid (Figure 4b). The average Raman spectra, derived from over 20 measurements for each sample, revealed A^1^g to A^2^_g_ intensity ratios of 0.719 for BPNSs and 0.716 for MV‐BPNSs, indicating a minimal oxidation rate throughout the MV‐BPNS hybrid preparation.^[^ ^39^ ^]^

**Figure 5:**
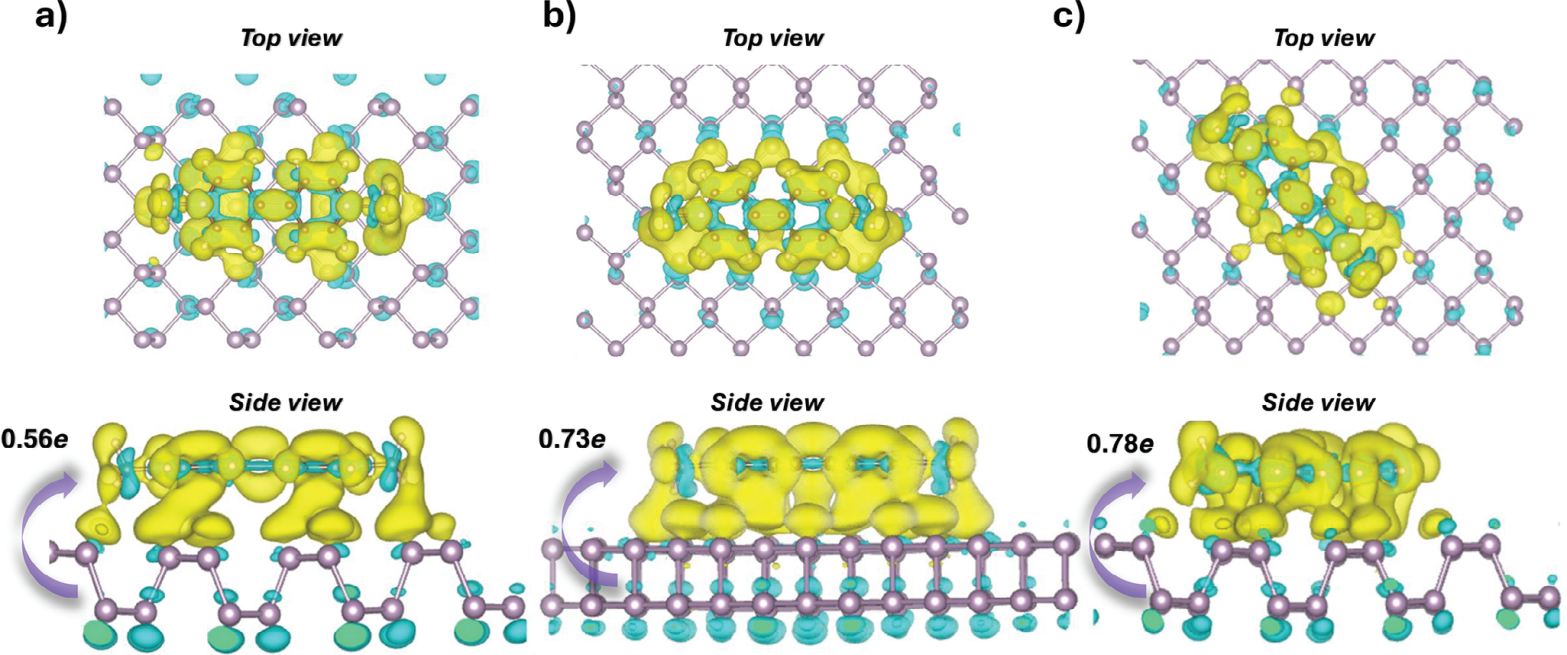
Charge difference plots of the MV molecules on top of a single‐layer BPNS surface. a) perpendicular to the ridge, b) parallel to the ridge, c) diagonal to the ridge. Orbital overlap scenarios for the MV‐BPNS hybrids in each direction are shown as the side view located under each consecutive illustration. The yellow region represents areas of electron accumulation, while the cyan region indicates areas of electron depletion.

To predict the properties and interaction of the viologen derivatives with BPNSs, density functional theory (DFT) studies were employed. The energy levels of the highest occupied molecular orbitals (HOMO) and lowest unoccupied molecular orbitals (LUMO) were calculated. The HOMO levels were determined to be −2.65 and −2.63 eV for MV and MV_2_, respectively, while the LUMO levels were found to be −0.94 and −1.25 eV for the same. The HOMO‐LUMO gaps were calculated to be 1.7 and 1.38 eV for MV and MV_2_, respectively. The normalized adsorption energies were calculated for MV (**Table** **1**) in the direction parallel, perpendicular and diagonally to the ridges of BPNSs (MV_2_‐BPNSs: Table S1, Supporting Information). For the MV_2_‐BPNS hybrid, the normalized adsorption energy value was found to be lower (−0.0472 eV) than that of the MV‐BPNS hybrid (−0.0518 eV), which explains the slower precipitation speed of MV_2_‐BPNS upon mixing (Table 1). DFT calculations also predicted partial charge transfer processes, with the extent of charge transferred from the BPNSs to the viologen derivatives measured at 0.73*e* and 1.75*e* for the MV‐BPNS and MV₂‐BPNS hybrids, respectively, along the parallel direction of the BPNS ridges (**Figure** **5**). DFT calculation results suggest the presence of possible cation‐induced dipole interactions between the MV molecules and BPNSs. Further calculations on the oxygen adsorption on the nanosheet surfaces revealed more information on the degradation mechanism. A previous study demonstrated that O_2_ readily dissociated on the black phosphorus surface.^[^ ^40^ ^]^ The adsorption of O₂ at various sites under MV coverage was calculated, revealing that O₂ similarly underwent spontaneous dissociation into two oxygen atoms (Figure S7, Supporting Information). As a result, the free energy of adsorption of the O atom was employed as the criterion for evaluating the ease of BPNS oxidation in all subsequent analyses. The Gibbs free energy for the oxygen atom adsorption was calculated for the surfaces of pure BPNSs, MV‐ and MV_2_‐BPNS hybrids. Interestingly, the calculated adsorption free energy increased by 0.04 and 0.02 eV for the MV‐ and MV_2_‐BPNS hybrids, respectively, compared to the pure BPNSs (Figures S8–S11, Supporting Information). This suggests that oxygen adsorption is more favorable and likely to occur spontaneously on pure BPNSs, whereas it is comparatively less probable on the MV‐BPNS hybrids. The calculation, however, did not account for the excitonic pairs generated on the nanosheets under light exposure or the charged superoxide radical anion species formed through electron transfer from the conduction band of the nanosheets to molecular oxygen.

**Table 1:** Total and average adsorption energy of the MV‐BPNSs hybrids along different directions from the ridges of BPNSs calculated by DFT.

Materials [MV‐BPNSs]	E_ads_ [eV]	Number of atoms [X]	E_ads_/X [eV]
Perpendicular	−1.45	28	−0.0518
Parallel	−1.47	28	−0.0524
Diagonal	−1.44	28	−0.0516

**Figure 6:**
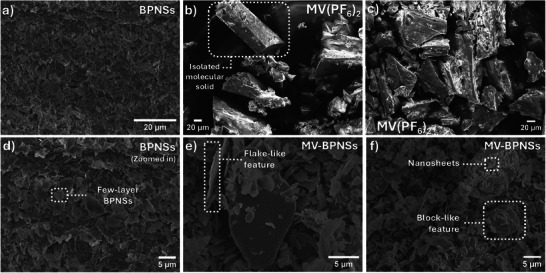
SEM images of a) BPNSs, b,c) MV(PF_6_)_2_, d) BPNSs (zoomed in) and e,f) the MV(PF_6_)_2_‐BPNS hybrids.

SEM was performed to investigate the surface morphology of the bare BPNSs and the viologen‐BPNS hybrids. To compare the hybrids, SEM imaging of the viologen derivatives were also recorded. Viologen derivatives exhibited a 3D morphology characterized with distinct solid, block‐like features (**Figure** **6**b,c), with rod‐like structures observed for MV (Figure 6b). The bare BPNSs showed flakes of nanosheets (Figure 6a–d), which was preserved in the MV‐BPNS hybrid (Figure 6e,f) along with large solid features scattered throughout the samples, indicating that the noncovalent functionalization process minimally impacts the overall morphology. The scale bars in Figure 6b,c were deliberately kept large to highlight the difference in aspect ratios between the molecular 3D features and the nanosheets' dimensions.

**Figure 7:**
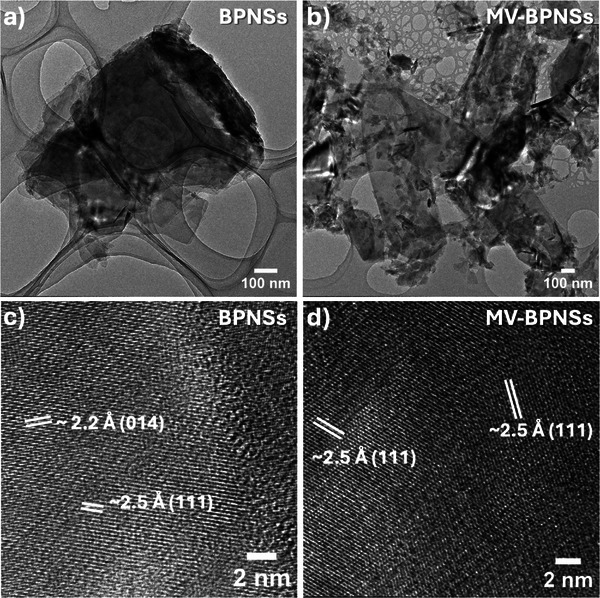
TEM and HR‐TEM images of a,c) BPNSs and b,d) the MV(PF_6_)_2_‐BPNS hybrids.

Furthermore, a closer look on the exfoliated and the functionalized BPNSs through transmission electron microscopy (TEM) and high‐resolution TEM (HR‐TEM) uncovered information on the lattice parameters of the sheets. **Figure** **7**a shows the morphology of a few‐layer BPNS, highlighting its structural features. The lattice fringes for the (111)^[^ ^41^ ^]^ plane and the (014) plane^[^ ^24^ ^]^ can be observed for the bare BPNSs with d‐spacings of ≈2.5 and 2.2 Å, respectively (Figure 7c). The (111) plane of BPNSs can be observed with the d‐spacing value of 2.5 Å in the MV‐BPNS hybrids, suggesting the unaffected crystallinity of BPNSs through the non‐covalent functionalization process (Figure 7d). It is worth mentioning that the MV‐BPNS hybrid displayed an aggregated morphology (Figure 7b; Figure S5b,c, Supporting Information), consistent with observations from UV titration and large‐scale sample preparation for IR, Raman, and XPS measurements. In contrast to this observation, the MV_2_‐BPNS hybrid did not show an aggregated morphology (Figure S12c, Supporting Information) as the MV_2_ molecules passed through the TEM grid due to their lower adsorption energy and slower aggregation kinetics.^[^ ^42^ ^]^

**Figure 8:**
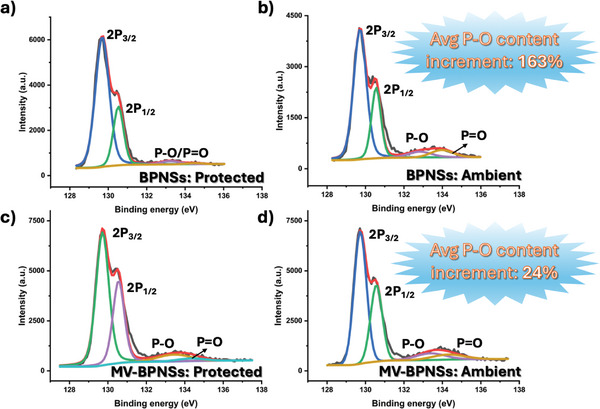
P2p XPS spectra of a,b) BPNS under protected and ambient conditions. c,d) MV‐BPNS hybrids under protected and ambient conditions.

**Figure 9:**
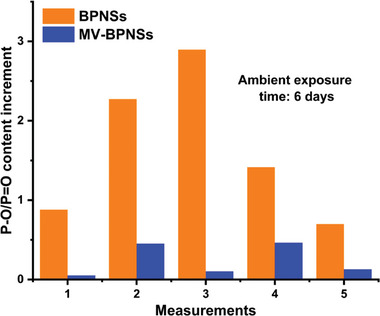
A comparison of the extent of oxidation between BPNSs and the MV‐BPNS hybrids in various measurements over 6 days of ambient exposure.

The ambient stability of the BPNSs and the hybrid materials were compared using X‐ray photoelectron spectroscopy (XPS), as shown in **Figure** **8**. A control experiment was conducted by storing pristine BPNSs and MV‐BPNS hybrids under nitrogen atmosphere for 6 days, while identical samples were kept under ambient conditions for the same duration. This procedure was repeated multiple times to ensure consistency.^[^ ^43^ ^]^ The core‐level P2p intensities were plotted against binding energy, revealing four fitted peaks for BPNSs under protected conditions at 129.69, 130.53, 133.23, and 134.47 eV (Figure 8a, **Table** **2**), corresponding to P2p_3/2_, P2p_1/2_, P─O and P═O, respectively. In the MV‐BPNS hybrid under protected conditions, the respective P signals were observed at 129.62, 130.46, 133.35, and 134.24 eV (Figure 8c, Table 2). The P2p intensity of P‐F for MV‐BPNSs could not be traced due to the low requirement of the MV molecule to form the hybrid, however, the molecular signal of N1s can still be traced in both the survey scan and the high‐resolution spectra (Figures S13 and S14, Supporting Information). For BPNSs and MV‐BPNSs kept under ambient conditions, the P2p_3/2_, P2p_1/2_, P─O and P═O peaks can be observed at 129.71, 130.55, 132.83, 133.97 eV and 129.73, 130.57, 133.39, 134.30 eV (Figure 8b–d and Table 2). For the samples stored under ambient conditions, a significant increase in the P─O/P ═O content was observed in the BPNSs (163%, Figure 8b). In contrast, the average increase in the P─O/P═O content for the MV‐BPNS hybrid was significantly lower (24%, Figure 8d). Repeated measurements confirmed a higher degree of oxidation in the BPNSs compared to the MV‐BPNS hybrids under similar experimental conditions, as shown in **Figure** **9**. However, the MV‐BPNS hybrid exhibited minimal degradation, showing the least change in the P─O/P═O content. Additionally, based on the atomic ratio between phosphorus and nitrogen in the XPS measurement, the noncovalent functionalization degree for the MV‐BPNS hybrid is calculated to be every ≈21 phosphorus atoms having one MV molecule. The proposed mechanism for the enhanced ambient stability of the hybrid may stem from a decrease in surface electron density of the BPNSs, likely due to encapsulation by the MV molecules. This encapsulation could effectively block oxygen and water molecules from accessing the BPNS surface.

**Table 2:** P2p binding energies of BPNSs and MV‐BPNS in XPS measurements.

Binding energy	BPNS‐P [eV]	BPNS‐A [eV]	MV‐BPNS‐P [eV]	M‐BPNS‐A [eV]
P2p_3/2_	129.69	129.71	129.62	129.73
P2p_1/2_	130.53	130.55	130.46	130.57
P−O	133.23	132.83	133.35	133.39
P = O	134.47	133.97	134.24	134.30

P‐protected, A‐ambient.

Another viologen derivative (MV_2_(PF_6_)_4_: Scheme S1 (Supporting Information) was also employed to address the ambient stability issues associated with BPNSs. Titrimetric studies using UV–vis absorption spectroscopy suggests the interaction between the MV_2_ molecules and BPNSs, where the absorption peak of MV_2_ was observed to be shifted from 287 to 291 nm (Figure S6, Supporting Information), upon the gradual addition of the MV_2_ molecule (1 mg mL^−1^) to a fixed concentration of BPNSs (1.7 mL of 0.118 mg mL^−1^ dispersion). However, the precipitate formation observed in this experiment did not translate effectively to large‐scale sample preparation, likely due to slower aggregation kinetics at higher concentrations and the potential presence of a dynamic adsorption‐desorption process. ATR‐IR measurements (Figure S9, Supporting Information) also showed a shift in the C–H out‐of‐plane bending mode in the hybrid from 818 to 837 cm^−1^ referring to the possible interactions between the MV_2_ molecules and BPNSs. SEM images showed flake‐like features of BPNSs alongside occasional scattered bulk features. The flake features of BPNSs in the hybrids were further confirmed by a d‐spacing of 2.2 Å for the (014) plane (Figure S12, Supporting Information) observed in the HR‐TEM measurements, with no distortion from aggregation, likely owing to the lower adsorption energy and slower aggregation kinetics. The ambient stability of the hybrids was also evaluated using XPS measurements (Figure S15, Supporting Information). However, due to the weak interaction and slow aggregation kinetics between the MV_2_ molecules and BPNSs, a high loading of MV₂ molecules is required to form the hybrid, leading to inhomogeneity across the hybrids. The peaks for the MV_2_‐BPNS hybrids stored in the protected conditions at 129.63, 130.47, 133.38, 134.28, 136.57 eV (Figure S15a, Table S2, Supporting Information) and 129.04, 129.88, 133.31134.28 and 136.18 eV (Figure S15c, Table S2, Supporting Information) can be assigned to P2p_3/2_, P2p_1/2_, P─O, P═O and P–F.^[^ ^24^, ^44^, ^45^ ^]^ The P–F peak was very intense in the case of the MV_2_‐BPNS hybrids, both in protected and ambient conditions, due to their weak interactions, high loading amount of MV_2_ and inhomogeneity (Figure S15, Supporting Information).^[^ ^46^, ^47^ ^]^ For MV_2_‐BPNSs in ambient condition, the P2p_3/2_, P2p_1/2_, P─O, P═O and P–F peaks can be observed at 129.84, 130.68, 133.38, 134.28, 136.73 eV (Figure S15c, Table S2, Supporting Information) and 129.74, 130.58, 133.33, 134.23, and 136.92 eV (Figure S15d, Table S2, Supporting Information). The increase in the total P─O/P═O content for the MV₂‐BPNS hybrid stored in ambient conditions varied significantly between samples, showing an approximate rise of 85% (from Figure S15a,b, Supporting Information) and up to 254% (from Figure S15c,d, Supporting Information), likely due to sample inhomogeneity.

## 3 Conclusion

In summary, two different viologen derivatives with 2 and 5 aromatic rings were successfully employed to passivate BPNS surface. The normalized adsorption energy was predicted using DFT calculations which directly correlates to the interactions of the viologen molecules with the BPNS surface and the interactions were supported by UV–vis absorption, FTIR and Raman spectroscopy. Contrary to our initial expectations, MV_2_ formed weaker hybrids with BPNSs compared to MV, likely due to its lower average adsorption energy and slower aggregation kinetics, despite having a higher charge. The ambient stability efficiency was investigated using XPS studies by comparing the MV‐BPNS hybrid to the pristine BPNSs in both protected and ambient conditions. BPNSs exhibited a higher oxidation tendency under aerobic conditions, even in their aggregated form, while the MV‐BPNS hybrid showed minimal oxidation, consistent with DFT‐based predictions, suggesting it as a potentially effective strategy for stabilizing BPNSs. Moreover, the non‐covalent functionalization approach maintains a low degree of modification, as confirmed by XPS results, ensuring that the intrinsic properties of BPNSs remain largely intact. In contrast, the MV_2_‐BPNS hybrid showed inhomogeneity, resulting in an inconclusive protective effect. As this appears to be a novel observation, it represents a significant finding that warrants communication to the broader research community. This study introduces a promising approach to enhancing the stability of BPNSs, making them more resistant to decomposition and potentially useful in energy storage systems, such as batteries and supercapacitors.

## 4 Experimental Section

### Chemicals and Materials

Black phosphorus (BP) was purchased from Smart Elements (≈99.998%) and methyl viologen dichloride was purchased from Sigma Aldrich (98%). The bulk BP and solvents were always kept inside in a glovebox. Transfer of solvents was carried out in a glovebox or nitrogen atmosphere. The solvent acetonitrile, dichloromethane and methanol were anhydrous and were purchased from Sigma–Aldrich. All the solvents were deoxygenated before the experiments unless otherwise mentioned.

### Characterizations

Raman analysis was performed on a WITec alpha300 R confocal Raman microscopy system (excitation at 532 nm). XPS analysis was carried out on a PHI 5000 VersaProbe III Scanning XPS Microprobe and the spectra were processed using the PHI multiPak software. TEM and HR‐TEM were performed on a FEI Tecnai T20 instrument at an acceleration voltage of 200 kV. ATR‐IR spectra were recorded using a PerkinElmer Frontier Infrared Spectrometer with a GladiATR, diamond crystal design. UV–vis absorption spectra were recorded on a Varian Cary 50 Bio UV–vis spectrophotometer. ^1^H NMR spectra of the molecules were recorded using a 600 MHz Bruker Avance NEO NMR spectrometer.

### Ion‐Exchange of Methyl Viologen

The reaction was performed by addition of excess ammonium hexafluorophosphate in a methyl viologen water solution (10 mg mL^−1^) and the precipitate by centrifugation was collected with additional washing using DI water.

### Preparation of BPNS

In a glove box, BP crystals (80 mg) were ground using a mortar and pestle and was transferred to a round bottom flask. The flask was filled with acetonitrile (60 mL) and was sonicated in 37 kHz for 24 h at 10–15 °C. The mixture was centrifugated at 10 °C at 3000 rpm for 30 min and the supernatant was collected separately to obtain single‐ and few‐layer BPNSs.

### Preparation of the MV‐BPNS and MV_2_‐BPNS Hybrids

For the MV‐BPNS hybrid, a BPNS dispersion in acetonitrile (5 mL, 0.14 mg mL^−1^) was mixed with a MV solution (100 µL, 1 mg mL^−1^) and the precipitate was isolated by centrifugation. To prepare the MV_2_‐BPNS hybrid, a BPNS dispersion in acetonitrile (5 mL, 0.14 mg mL⁻¹) was combined with a MV_2_ solution (500 µL, 1 mg mL⁻¹). However, due to the absence of precipitate formation—attributable to the slower aggregation kinetics and lower average adsorption energy—acetonitrile was evaporated under a nitrogen atmosphere, resulting in inhomogeneity within the MV_2_‐BPNS hybrid.

### Computational Methods

All first‐principles calculations were implemented using the Vienna ab initio simulation package (VASP).^[^ ^48^, ^49^ ^]^ The Perdew, Burke, and Ernzerhof (PBE) functional of generalized gradient approximation (GGA) served to approximate exchange‐correlation functions.^[^ ^50^, ^51^ ^]^ The projector‐augmented wave (PAW) method was used to represent the core‐valence electron interactions.^[^ ^52^, ^53^ ^]^ The cutoff energy of the plane‐wave basis was set to 400 eV. The threshold for the convergence in the self‐consistent field (SCF) was set to 10–5 eV and the ionic relaxation steps were stopped when the forces became smaller than 0.05 eV Å^−1^. The dispersion interactions were achieved using Grimme's D3 method.^[^ ^54^ ^]^ The BPNS model of the MV‐BPNS and MV_2_‐BPNS hybrids were constructed by (6 × 4) and (10 × 4) black phosphorus supercells. The Monkhorst‐Pack K point sampling mesh of 1 × 1 × 1 and 2 × 2 × 1 were chosen for geometry optimizations and electronic structure calculations, respectively. The vacuum space of the slabs was set as 18 Å to minimize the interaction between the periodic units. The ΔG was calculated from the equation: Δ*G* = Δ*E* + Δ*ZPE* − *T*Δ*S*, where Δ*E* refers to the electronic energy difference,  Δ*ZPE* and Δ*S* refer to the change of zero‐point energy and entropy at room temperature, respectively. *T* refers to 298.15 K. The ΔG_*O_ was calculated from the equation: $\Delta G_{*o}=G_{*o}-G_{*}-G_{H_{2}O}+G_{H_{2}}$. In the above equation, ΔG_*O_ denotes the adsorption free energy of ^*^O, G_*O_ refers to the free energy of the substrate with an adsorbed O atom, G^*^ represents the free energy of the substrate, and G_H2O_ and G_H2_ denote the free energies of the H_2_O and H_2_ molecules, respectively.

## Conflict of Interest

The authors declare no conflict of interest.

## Supporting information



Supporting Information

## Data Availability

The data that support the findings of this study are available from the corresponding author upon reasonable request.
