# Supplementary material for: Boosting 2D Black Phosphorus Ambient Stability: Noncovalent Functionalization Using Viologen Molecules
Source: Small. 2025 Mar 21;22(25):2410300. doi: 10.1002/smll.202410300 (PMC13137228; doi:10.1002/smll.202410300)
Supplement: Supplementary file 1 — Supporting Information [file SMLL-22-2410300-s001.pdf]

Supporting Information

Boosting 2D Black Phosphorus Ambient Stability: Noncovalent Functionalization using Viologen Molecules

*Ishan Sarkar[a], Cong Guo[b], Cheng Peng[a], Yu Wang[b], Yafei Li[b], Xiaoyan Zhang[a]**

I. Sarkar, C. Peng, Prof. X. Zhang
Department of Chemistry and Chemical Engineering
Chalmers University of Technology
Kemigården 4, SE-412 96 Göteborg, Sweden
E-mail: xiaoyan.zhang@chalmers.se

Dr. C. Guo, Prof. Y. Wang, Prof. Y. Li
Jiangsu Collaborative Innovation Centre of Biomedical Functional Materials, School of Chemistry and Materials Science, Nanjing Normal University, Nanjing, 210023 P. R. China

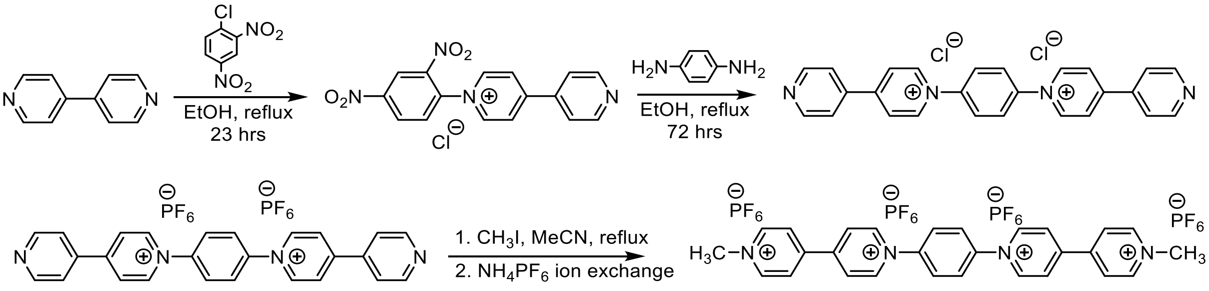

Scheme S1: Synthesis scheme for the MV_2-based viologen derivative.

Synthesis of 1-(2,4-dinitrophenyl)-[4,4'-bipyridin]-1-ium chloride (DNBP-Cl): 2,4-dinitrochlorobenzene (1 g, 4.9 mmol) and 4,4'-bipyridine (848.2 mg, 5.4 mmol) were dissolved in ethanol (25 mL). The mixture was heated under reflux for 24 h. The hot mixture was filtered, and the filtered cake was washed by acetone (2 × 80 mL). The solvent was evaporated and the remaining solid was recrystallized from C_2H_5OH/ether. The compound was obtained as a brown solid (0.45 g, 26%). Yield: 25.3% (448 mg), ^{1}H NMR (600 MHz, D_2O, δ): 9.40 (d, 1H), 9.26 (d, 2H), 8.95 (dd, 1H), 8.86 (d, 2H), 8.70 (d, 2H), 8.29 (d, 1H), 8.06 (d, 2H).

Synthesis of 1,1''-(1,4-phenylene)bis(([4,4'-bipyridin]-1-ium)) hexafluorophosphate ($MV_2(PF_6)_2$): 1-(2,4-dinitrophenyl)-[4,4'-bipyridin]-1-ium chloride (447.6 mg, 1.249 mmol) and *p*-phenylenediamine (64.23 mg, 0.594 mmol) were dissolved in ethanol (15 mL). The mixture was heated under reflux for 72 h. The solvent was evaporated and the remaining solid was washed by acetone (200 mL). The filter cake was collected and was dissolved in water and its hexafluorophosphate salt mixture was obtained by ion-exchange with NH_4PF_6 (excess). The pure compound was obtained as brown solid by recrystallization in MeCN/diethylether (1:3). Yield: 23.6% (91 mg), ^{1}H NMR (600 MHz, CD_3CN, δ): 9.11 (d, 4H), 8.93 (d, 4H), 8.58 (d, 4H), 8.12 (s, 4H), 7.93 (d, 4H).

Synthesis of 1',1'''-(1,4-phenylene)bis(1-methyl-[4,4'-bipyridine]-1,1'-diium) hexafluorophosphate ($MV_2(PF_6)_4$): In a sealed tube, 1,1''-(1,4-phenylene)bis(([4,4'-bipyridin]-

1-ium)) hexafluorophosphate (91 mg, 0.14 mmol) was dissolved in acetonitrile (5 ml) and to it methyl iodide (1 ml) was added. The mixture was heated to 80 °C and kept running overnight. The reaction was stopped afterwards, and the solvent was evaporated. The solid obtained was dissolved into water and its hexafluorophosphate salt was obtained by ion-exchange with NH_4PF_6 (excess). Yield: 62.2% (87 mg), ^{1}H NMR (600 MHz, CD_3CN, δ): 9.25 (d, 4H), 8.92 (d, 4H), 8.66 (d, 4H), 8.50 (d, 4H), 8.17 (s, 4H), 4.45 (s, 6H).

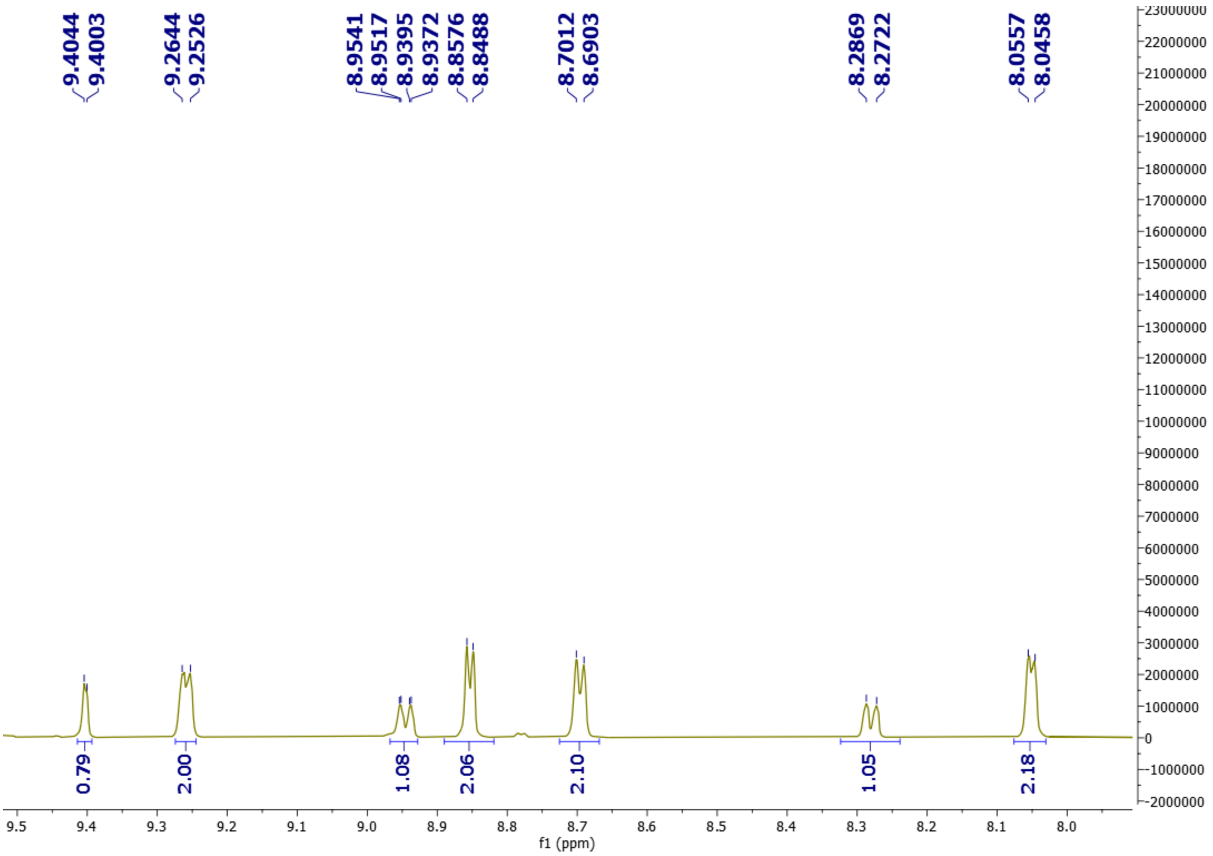

Figure S1. ^{1}H NMR spectrum of DNBP-Cl (D_2O, 600 MHz).

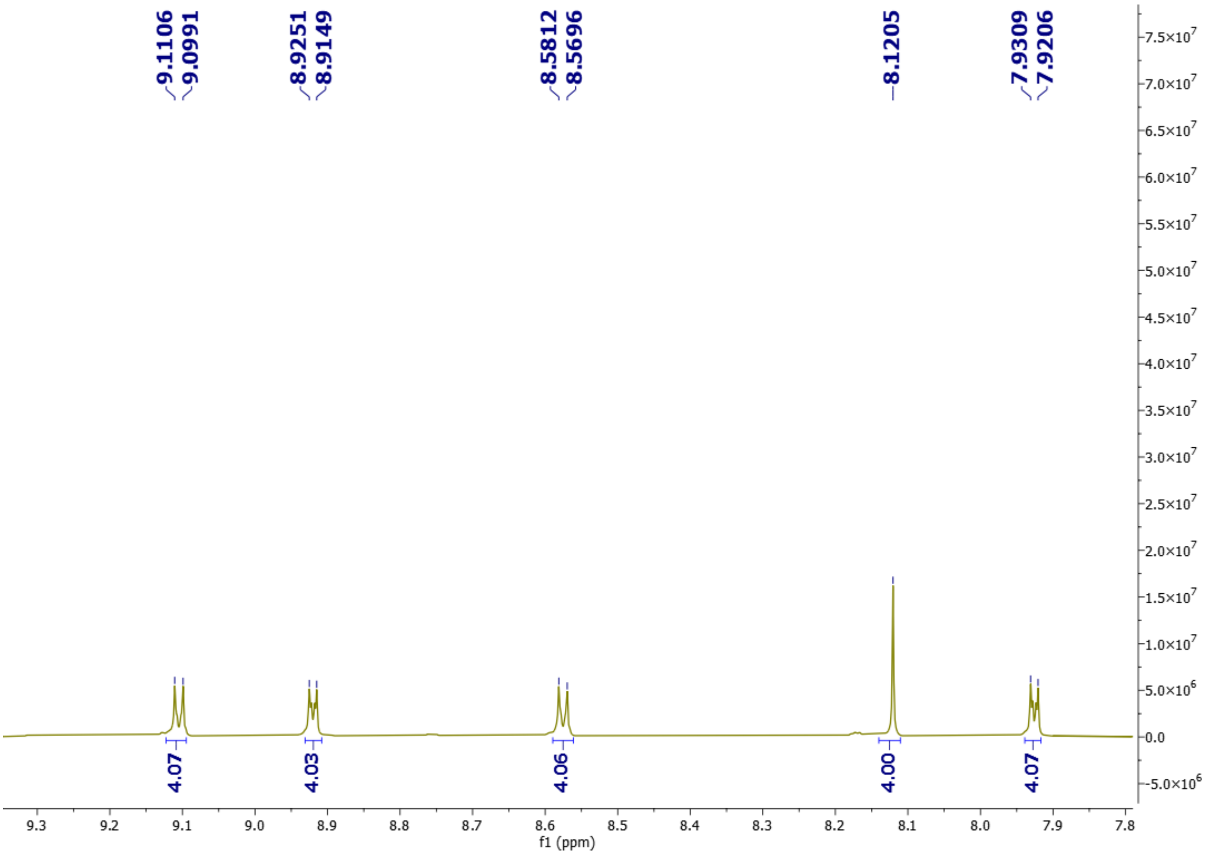

Figure S2. ^{1}H NMR spectrum of $MV_2(PF_6)_2$ (CD_3CN, 600 MHz).

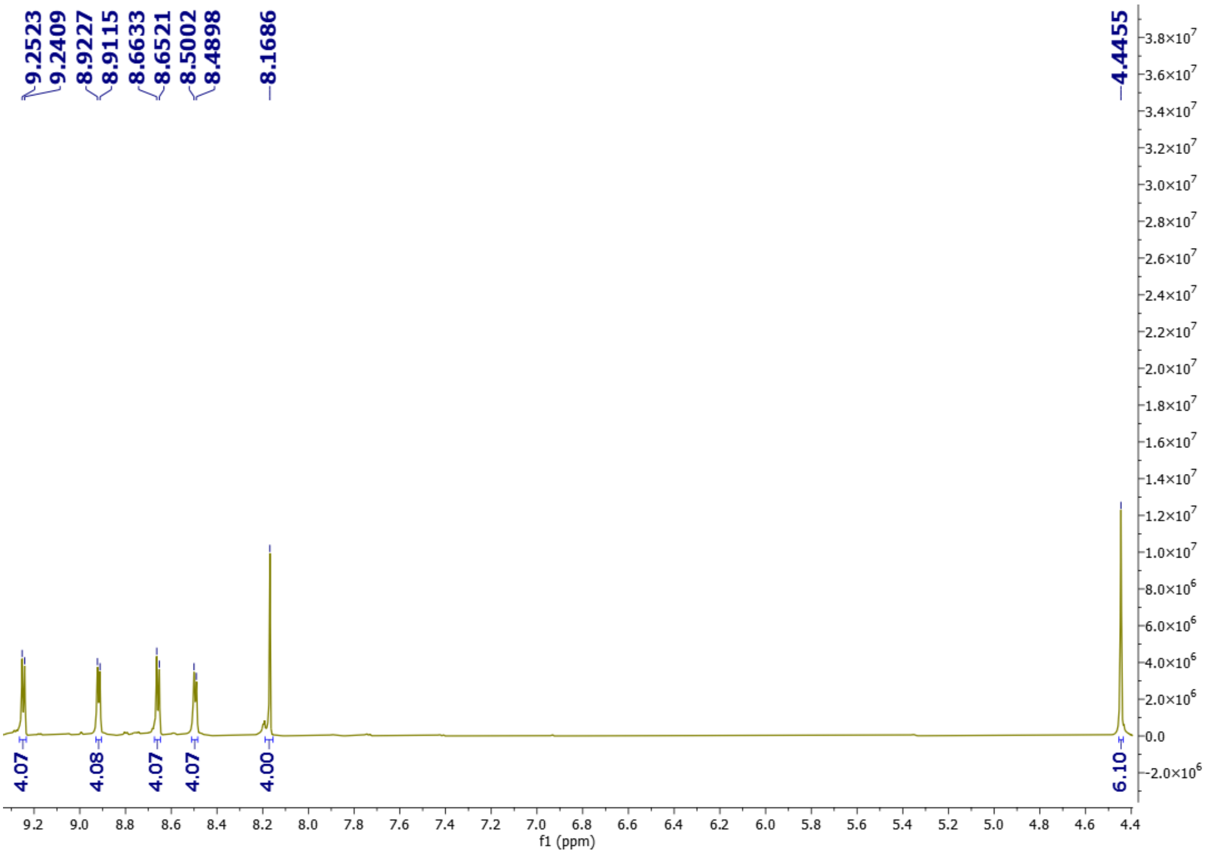

Figure S3. ^{1}H NMR spectrum of $MV_2(PF_6)_4$ (CD_3CN, 600 MHz).

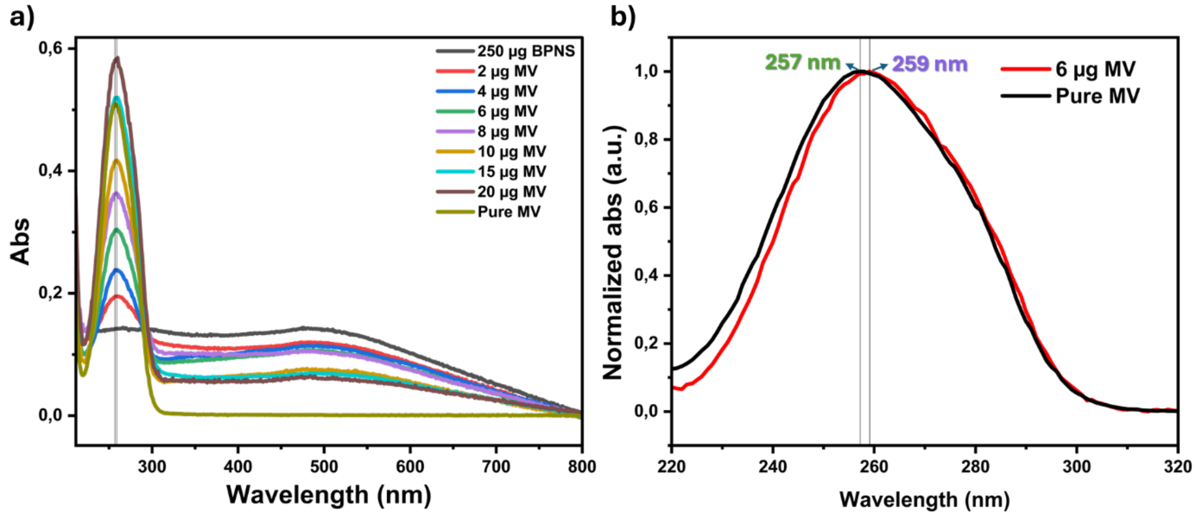

Figure S4. UV-vis absorption based titrimetric study between $MV(PF_6)_2$ and BPNSs, showing (a) full spectra of the hybrids compared to the pure molecular absorption (with zeroing) and (b) normalized absorption spectra showing the shift in peak absorbance.

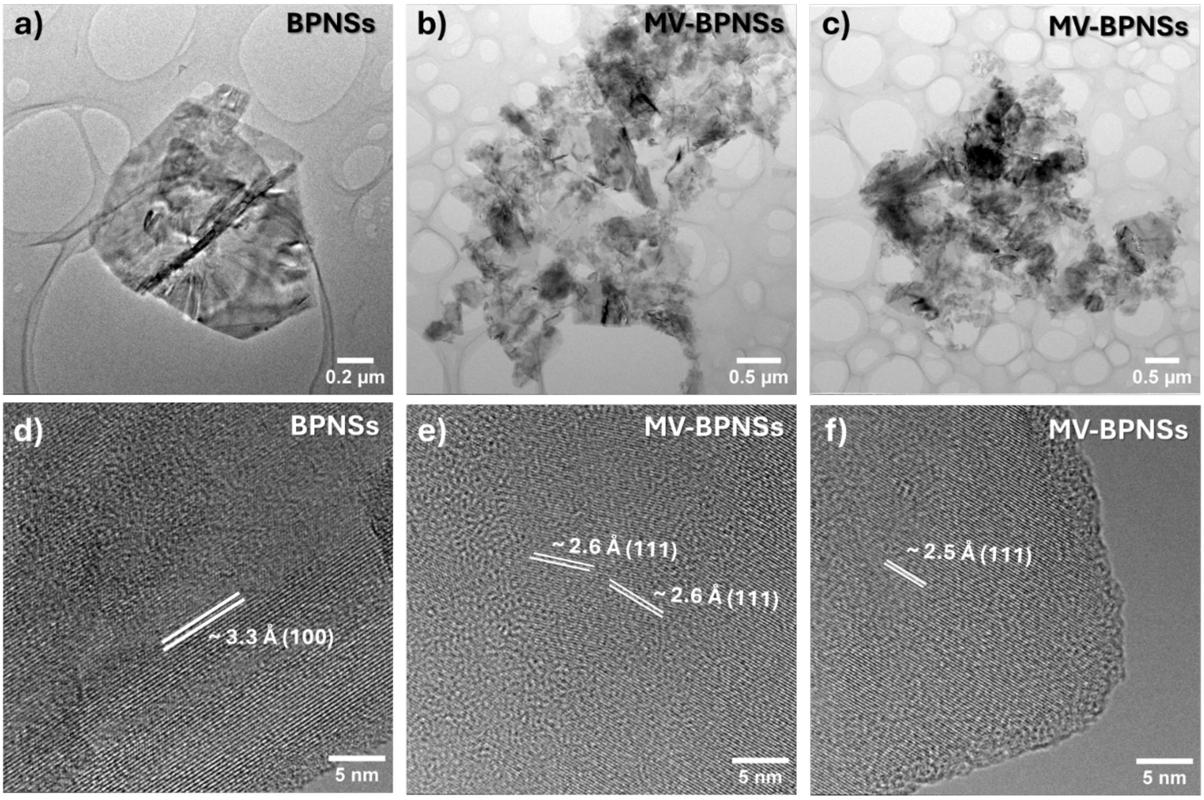

Figure S5. Additional TEM images of (a) BPNSs, (b,c) the MV-BPNS hybrids and HR-TEM images of (d) BPNSs showing the (100) plane with a d-spacing of approximately 3.3 Å and (e,f) the MV-BPNS hybrids showing the (111) planes with a d-spacing of approximately 2.6 Å.

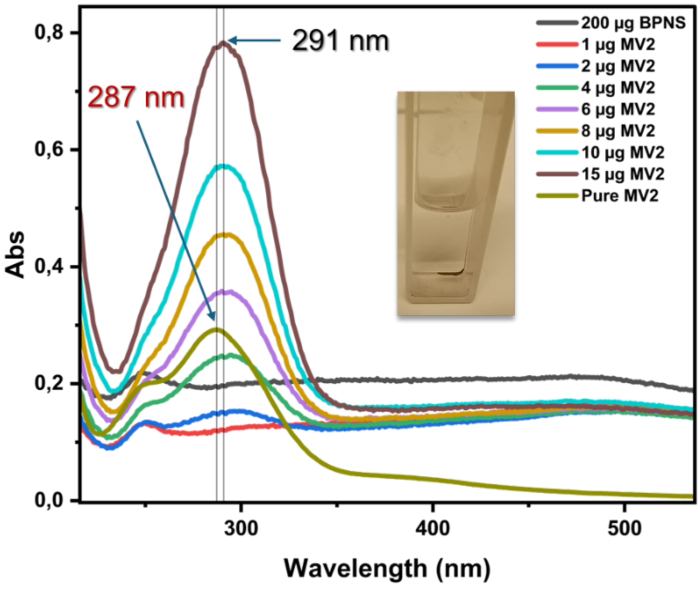

Figure S6. UV-vis absorption based titrimetric study between $MV_2(PF_6)_4$ and BPNSs, indicating the interaction between MV_2 and BPNSs at a low concentration level. The absorbance of $MV_2(PF_6)_4$ in acetonitrile (1.55 ml, 0.0045 mg ml^{-1}) was measured for comparison.

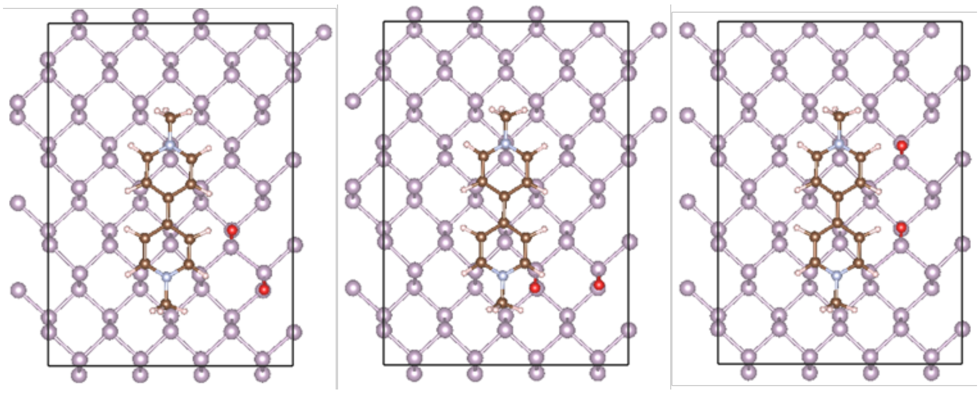

Figure S7. Structures of O_2 after adsorption at different sites on MV-BPNS.

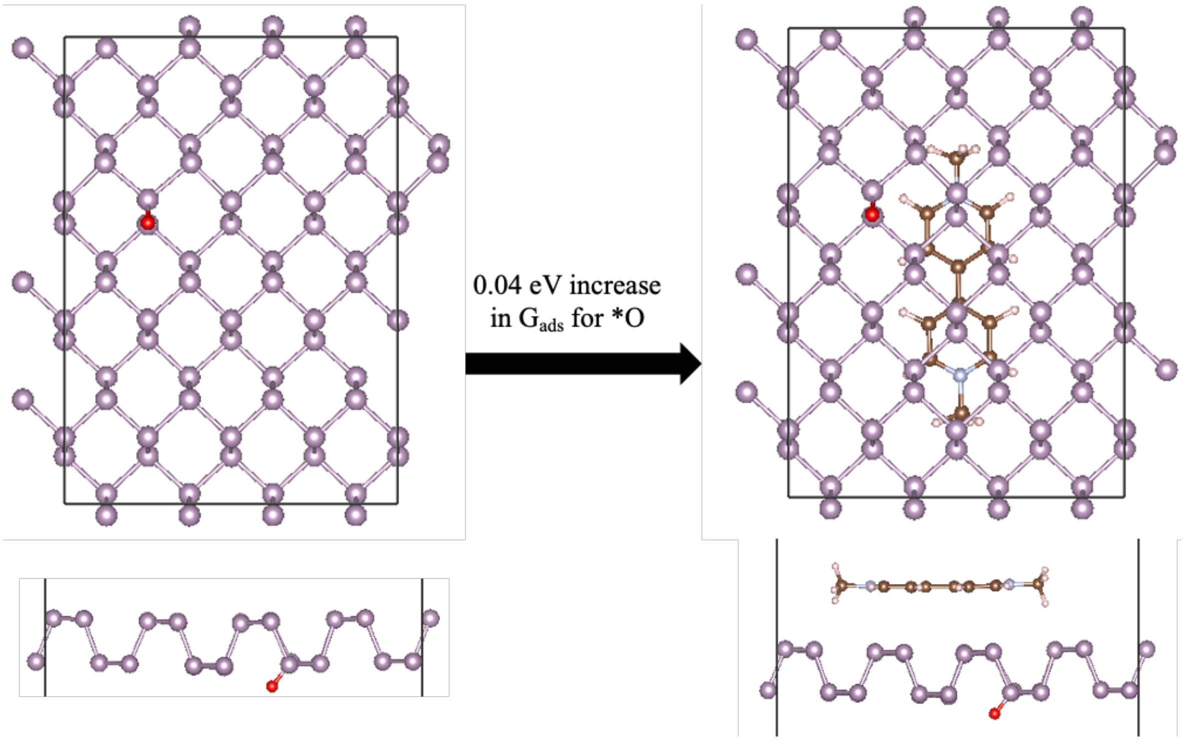

Figure S8. The calculated adsorption free energy increased by 0.04 eV for the MV-BPNS hybrid, compared to the pure BPNS.

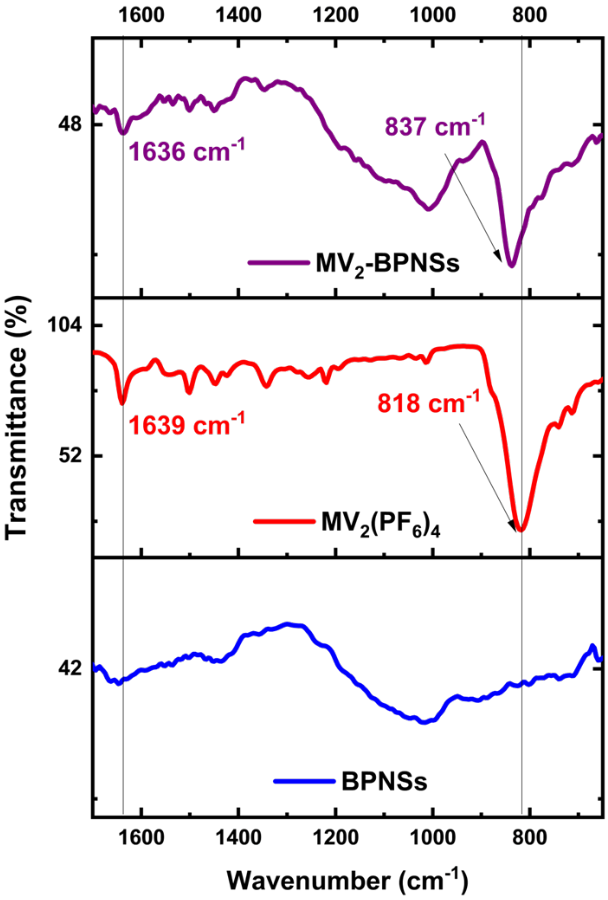

Figure S9. ATR-IR spectra of BPNSs, $MV_2(PF_6)_4$ and the MV_2-BPNS hybrid.

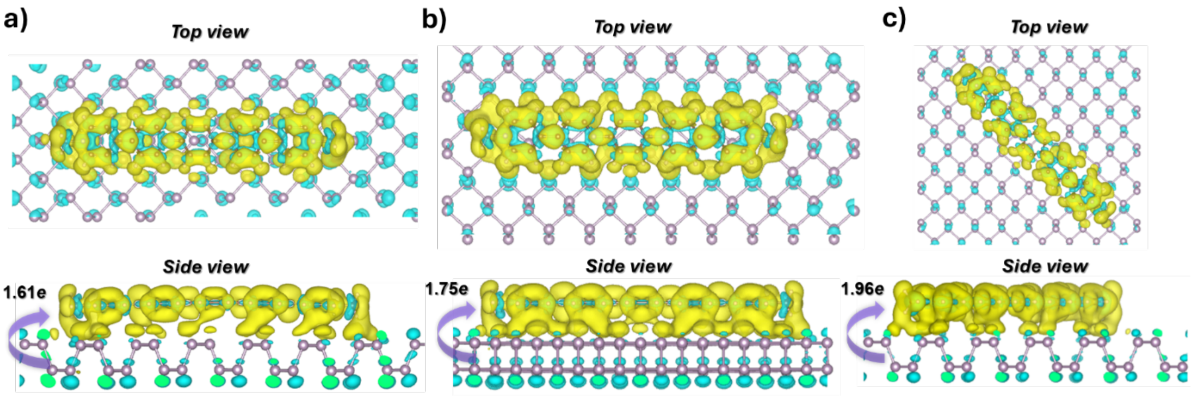

Figure S10. Charge difference plots of the MV_2 molecules on top of a single-layer BPNS surface: (a) perpendicular to the ridge, (b) parallel to the ridge, (c) diagonal to the ridge. Orbital overlap scenarios for the MV_2-BPNS hybrids in each direction are shown as the side view located under each consecutive illustration. The yellow region represents areas of electron accumulation, while the cyan region indicates areas of electron depletion.

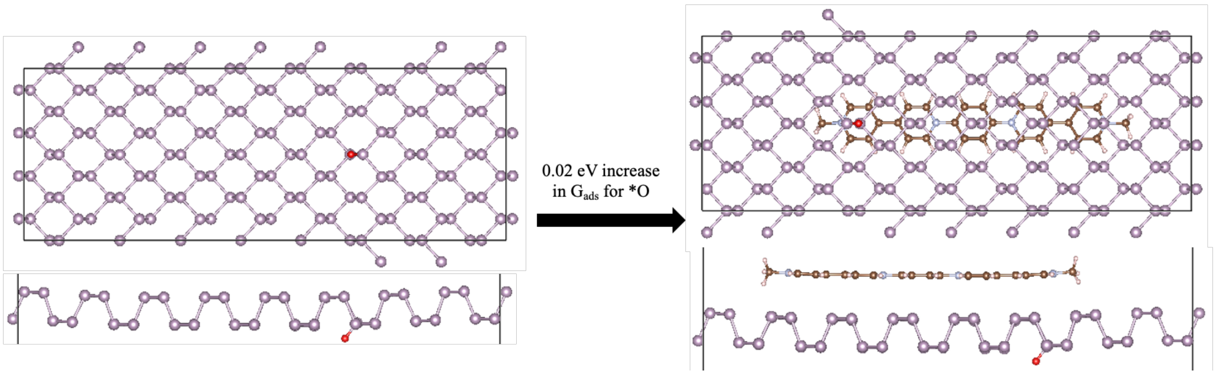

Figure S11. The calculated adsorption free energy increased by 0.02 eV for the MV_2-BPNS hybrid, compared to the pure BPNS.

Table S1. Total and average adsorption energy of the MV_2-BPNSs hybrids along different directions from the ridges of BPNSs calculated by DFT.

Materials [MV_2-BPNSs]	E_{ads} (eV)	Number of atoms (X)	E_{ads}/X (eV)
Perpendicular	−2.74	58	−0.0472
Parallel	−2.83	58	−0.0487
Diagonal	−2.67	58	−0.0460

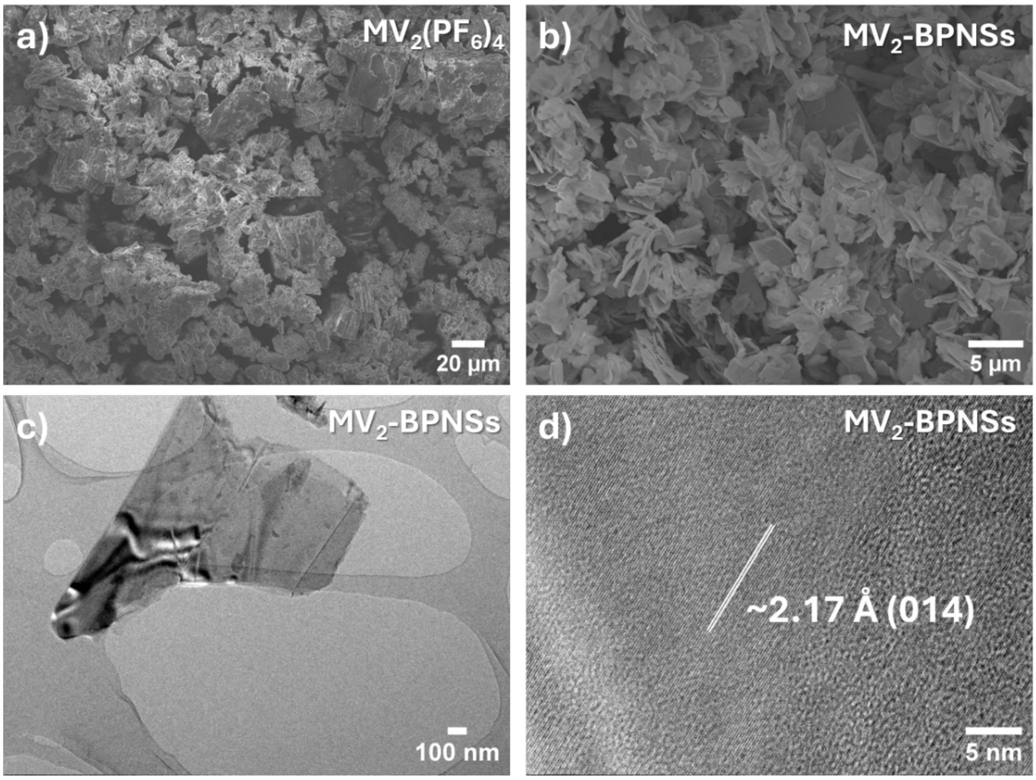

Figure S12. Electron microscopy images of the MV_2-BPNS hybrids (a) SEM image of $MV_2(PF_6)_4$, (b) SEM image of the $MV_2(PF_6)_4$-BPNS hybrid (zoomed in), (c) TEM image of the $MV_2(PF_6)_4$-BPNS hybrid and (d) HR-TEM image of the $MV_2(PF_6)_4$-BPNS hybrid. The MV_2-BPNS hybrid did not show an aggregated morphology as the MV_2 molecules might pass through the TEM grid due to the lower adsorption energy of the molecule and slower aggregation kinetics.

Table S2. P2p binding energies of the MV_2-BPNS hybrid in XPS measurements.

**Binding Energy**	**MV_2-BPNS-P1 (eV)**	**MV2-BPNS-P2 (eV)**	**MV_2-BPNS-A1 (eV)**	**MV_2-BPNS-A2 (eV)**
$P2p_{3/2}$	129.63	129.04	129.84	129.74
$P2p_{1/2}$	130.47	129.88	130.68	130.58
P−O	133.38	133.31	133.38	133.33
P=O	134.28	134.28	134.28	134.23
P-F	136.57	136.18	136.73	136.92

P-protected, A-ambient

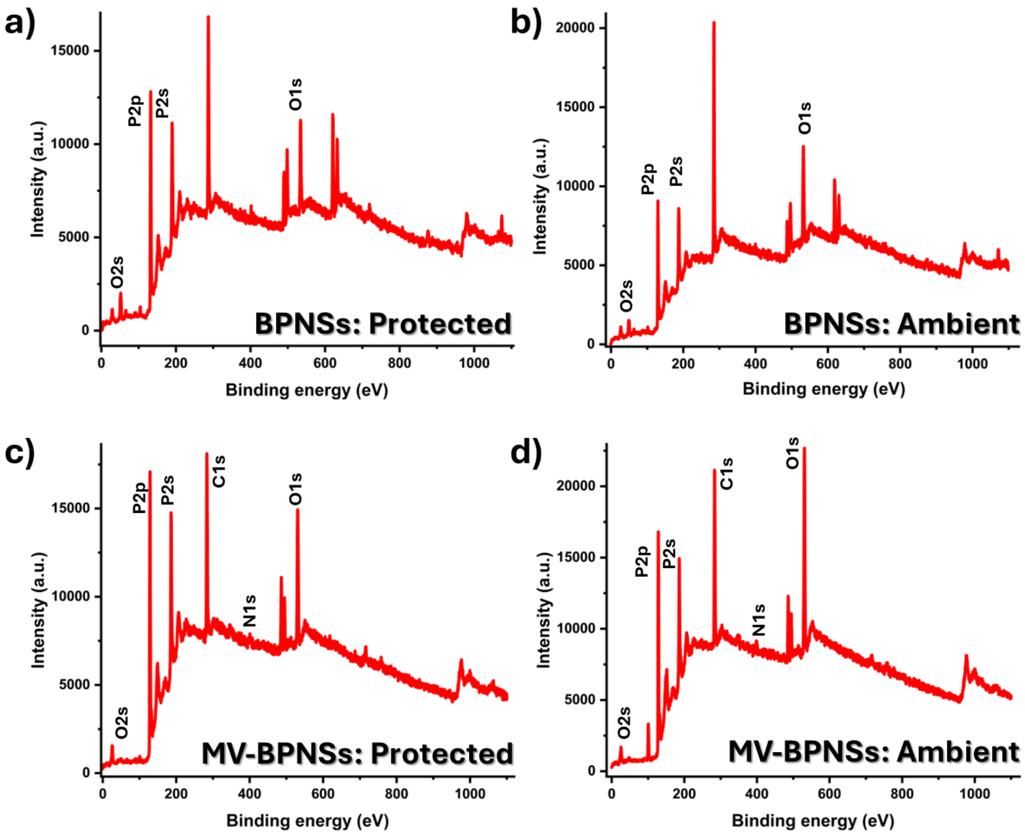

Figure S13. XPS: Survey spectra of (a) BPNSs in protected conditions (b) BPNSs in ambient conditions, (c) MV-BPNSs in protected conditions and (d) MV-BPNSs in ambient conditions.

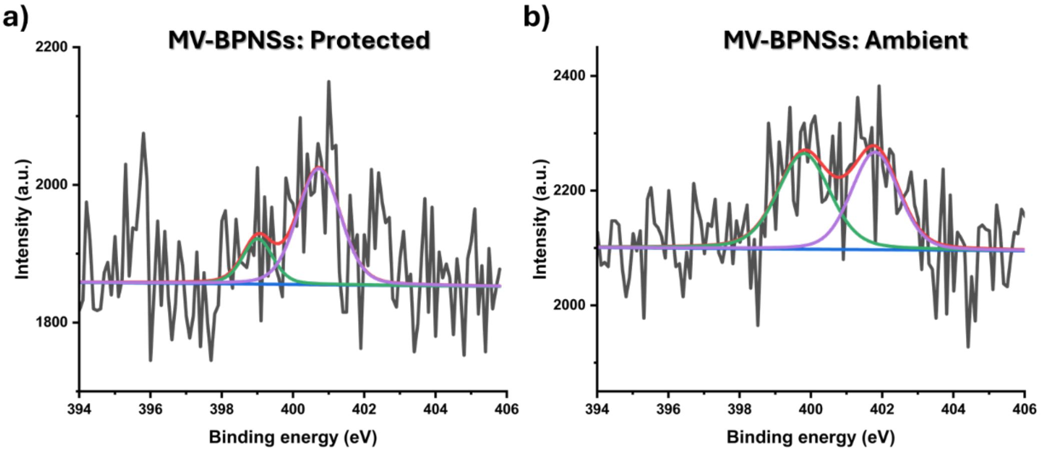

Figure S14. N1s XPS spectra of MV-BPNSs in (a) protected and (b) ambient conditions. The charged nitrogen (N^+) and radical cation ($N^{\cdot+}$) generated during X-ray exposure in the XPS chamber can be assigned to 400.7 eV and 399 eV for the protected hybrid and 401.8 eV and 399.8 eV for the hybrid in ambient condition.[S1] The sample preparation was performed in acetonitrile and the acetonitrile signal was not observed at 397.7 eV.[S2]

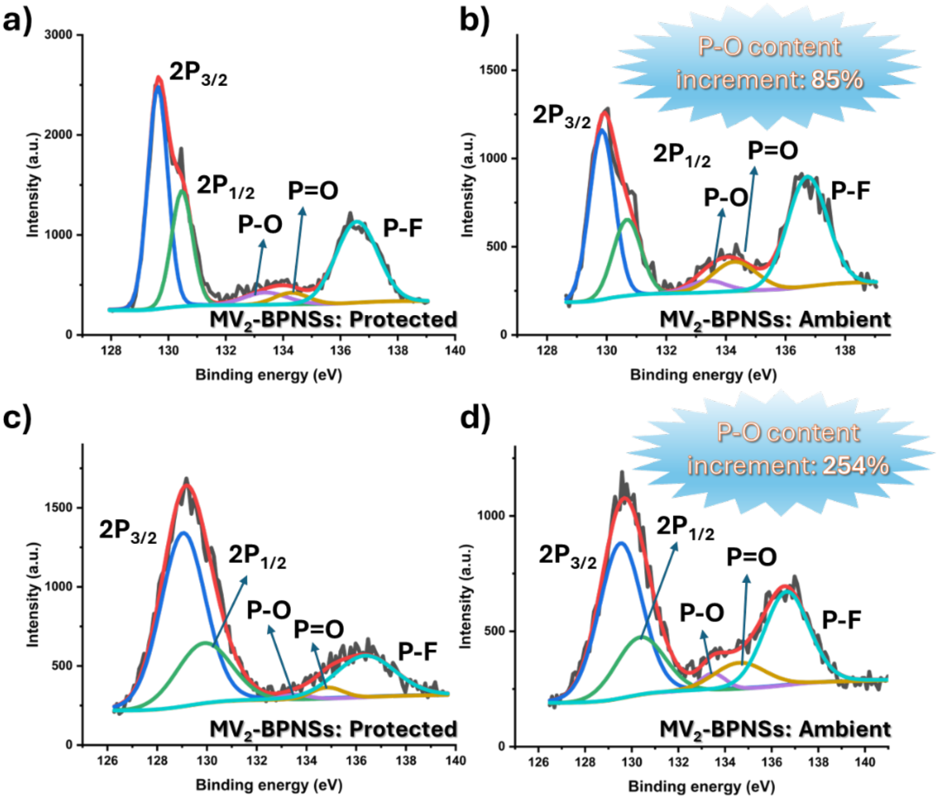

Figure S15. P2p XPS spectra of the MV_2-BPNS hybrid under (a,c) protected and (b,d) ambient conditions. The increase in the P-O/P=O content was compared under similar experimental conditions.

[S1]. Z. Wei, W. Shin, H. Jiang, X. Wu, W. F. Stickle, G. Chen, J. Lu, P. Alex Greaney, F. Du, X. Ji, *Nat. Commun.* **2019,** *10*, 3227.
[S2]. B. A. Sexton, N. R. Sexton, *Surf. Sci.* **1983,** *129*, 21-36.
